# Stigmatized by association: challenges for abortion service providers in Ghana

**DOI:** 10.1186/s12913-016-1733-7

**Published:** 2016-09-10

**Authors:** Patience Aniteye, Beverley O’Brien, Susannah H. Mayhew

**Affiliations:** 1 School of Nursing, College of Health Sciences, University of Ghana, P.O.BOX LG 43, Legon, Ghana; 2 Edmonton Clinic Health Academy (ECHA), Faculty of Nursing, 11405-87 Ave, 3rd Fl, Edmonton, Alberta T6G1C9 Canada; 3 Faculty of Public Health and Policy, London School of Hygiene & Tropical Medicine, London, WC1H 9SH UK

**Keywords:** Stigma, Abortion, Skilled providers, Ghana, Qualitative research

## Abstract

**Background:**

Unsafe abortion is an issue of public health concern and contributes significantly to maternal morbidity and mortality globally. Abortion evokes religious, moral, ethical, socio-cultural and medical concerns which mean it is highly stigmatized and this poses a threat to both providers and researchers. This study sought to explore challenges to providing safe abortion services from the perspective of health providers in Ghana.

**Methods:**

A descriptive qualitative study using in-depth interviews was conducted. The study was conducted in three (3) hospitals and five (5) health centres in the capital city in Ghana. Participants (*n* = 36) consisted of obstetrician/gynaecologists, nurse-midwives and pharmacists.

**Results:**

Stigma affects provision of safe-abortion services in Ghana in a number of ways. The ambiguities in Ghanaian abortion law and lack of overt institutional support for practitioners increased reluctance to openly provide for fear of stigmatisation and legal threat. Negative provider attitudes that stigmatised women seeking abortion care were frequently driven by socio-cultural and religious norms that highly stigmatise abortion practice. Exposure to higher levels of education, including training overseas, seemed to result in more positive, less stigmatising views towards the need for safe abortion services. Nevertheless, physicians open to practicing abortion were still very concerned about stigma by association.

**Conclusions:**

Stigma constitutes an overarching impediment for abortion service provision. It affects health providers providing such services and even researchers who study the subject. Exposure to wider debate and education seem to influence attitudes and values clarification training may prove useful. Proper dissemination of existing guidelines and overt institutional support for provision of safe services also needs to be rolled out.

## Background

The World Health Organization (WHO) defines unsafe abortion as a procedure for terminating a pregnancy that is performed by an individual lacking the necessary skills, or in an environment that does not conform to minimal medical standards, or both [1]. In Ghana, unsafe abortion contributes significantly to maternal morbidity and mortality, especially among adolescents [2]. Ghana’s maternal mortality rate is considered poor at 319 per 100,000 live births [3]. While globally unsafe abortion is thought to contribute to between 8 or 12 % of maternal deaths, in Ghana it is estimated that up to 30 percent of all maternal deaths may result from unsafe abortion, making it a leading direct cause of maternal mortality [4].

In 1985, a group of doctors in Ghana spearheaded an amendment of the Criminal Code on abortion. This led to an amendment in the law in 1985 with the following provisions: 1) Abortion is illegal unless carried out in a hospital or designated clinic by a registered medical practitioner or gynaecological specialist; 2) Abortion is permitted when continuation of the pregnancy will pose serious risk to the life of the pregnant woman or injury to her physical or mental health; 3) Abortion is also permitted where the pregnancy is the result of rape, defilement of a female idiot or incest or where there is substantial risk of a serious physical abnormality or disease in the foetus [5]. (PNDC, 1985). The abortion law did not explicitly mention gestational age limits or methods to be employed for the procedure. The law states that “medical practitioners” are permitted to provide surgical abortions, for both spontaneous and induced abortion, in designated health facilities; they also provide post-abortion care. Problematically, it does not clearly define who “medical practitioners” are and it was widely interpreted as meaning only doctors.

Not until 2006 did the Ghana Health Service produce operational protocols which included unsafe abortion management as well as abortion and post-abortion care procedures [6, 7]. This operational document clarified that medical practitioners (doctors), obstetricians nurse-midwives, community health officers and medical assistants with midwifery training are allowed to provide either medical or surgical abortions, at different levels of the health care system (community, sub-district, district, regional and national). Nurse/midwives and community health officers are allowed to perform medical abortions with pregnancies less than nine (9) weeks. Where pregnancies are over nine (9) weeks, these cadres are only allowed to conduct medical abortion at levels where doctors are available to supervise them (e.g. district level). In Ghana, abortions are carried out through manual vacuum aspiration (MVA), medical abortion, dilation and curettage (D&C) and dilation and evacuation (D&E). Only medical practitioners and obstetricians practise D&C and D&E. The protocols have not been widely disseminated. Although this study took place after the protocols had been published, many health providers working in obstetric and gynaecological units, especially nurse-midwives, were not aware of the contents.

Despite the publication of the abortion protocols, which included post-abortion care provisions, there was very limited availability of abortion services at the time of the study [2, 7]. Private hospitals are known to carry out safe abortions (mainly MVA, though increasingly medical abortion) for high fees, some NGOs like Marie Stopes International and Ipas provide services at a reduced fee and offer both MVA and medical abortion (depending on gestation), but are not widely known. Abortions are not usually openly available in public health facilities for fear of prosecution though they are provided by doctors in a clandestine manner and often labelled as ‘incomplete’ (spontaneous abortions) or diagnostic dilatation and curettage. Some health and paramedical staff, including nurses (especially male nurses), and some doctors also carry out abortions clandestinely in private undesignated premises, but these are against the law and are liable to face prosecution, although prosecutions are few and rare. Ghanaian women who seek abortion may do so from pharmacy shops (buying misoprostal, although at the time of the study it had not been formally licenced but was undergoing country trials), private hospitals, some public hospitals and other areas, such as clandestinely in the ‘providers’ homes and usually for high fees [8]. Information from an international NGO (Ipas) reports that the cost of abortion care in Ghana ranges from US$30 toUS$40 in public health facilities [9]. This cost is high because few doctors provide services and those who do consider it professionally risky (because of stigma associations) to perform abortion. The consequence of high fees is to make this service inaccessible for poor women and especially adolescents [9]. The fees women pay for procuring abortions are unofficial; they are amounts women are estimated to pay in practice. They are not fees stipulated by the Ghana Health Service or Ministry of Health. The abortion providers usually charge women arbitrarily, based on the gestational age of the pregnancies. Due to the secrecy surrounding abortion provision at the time of the study in both public and private health facilities, no official fees were available.

A clear barrier to accessing abortion services is that abortion is frequently characterized by intense stigma and shame associated with criminalization of the procedure as well as moral and religious condemnation [10–14]. Stigma is an attribute of a profoundly discrediting nature that marks or taints an individual as one who should be socially avoided [15]. Link and Phelan [16] describe stigma as a multifaceted process that operates at multiple levels. Stigma by association or courtesy stigma refers to family, friends and caregivers of women procuring abortion who are discredited merely because of their connection to a stigmatized person [17, 18]. In addition to mental illness, HIV/AIDS, tuberculosis, leprosy, obesity and abortion are phenomena that are commonly stigmatized [19–23]. Individuals who have had an abortion, performed one, or become involved in abortion controversy are vulnerable to stigmatization. These societal attitudes may have ramifications for patients, health providers and even researchers who work to facilitate the safety and comfort of women who seek/experience abortion.

Problems are best solved when different dimensions are investigated and understood. In Ghana, little evidence was found regarding abortion stigma, especially “stigma by association” encountered by providers and researchers [24]. Martin et al. [24] in their study on abortion stigma (from the perspectives of some Ghanaian doctors), found the operation and manifestations of abortion stigma in Ghana as multi-dimensional. The pervasive social stigma influenced the content and implementation of the abortion law and policy; the relatively liberal but ambiguous law made its interpretation and application problematic. Availability and access to safe abortion services was limited leading to clandestine, unsafe abortions with their attendant complications including maternal mortality. Women procuring abortions and doctors providing the services were highly stigmatized in Ghanaian society with untold ramifications, yet there was paucity of research to elucidate the heavy burden stigma places on women, doctors and researchers. The aim of this paper is to critically analyse health providers’ perspectives on challenges to providing safe abortion services in Ghana in order to provide a better and deeper understanding of how stigma is experienced and what might be done to reduce it. Investigating the impact of stigma, especially on different cadres of health providers would shed more light on the observed attitudes of providers towards abortion services and training as well as availability, access and safety of the services. The lead author of this manuscript, a health provider (nurse/midwife) and researcher of the subject also shares her lived experiences of stigma of association, adding a novel dimension of researchers’ experiences of abortion stigma. Furthermore, deeper socio-cultural dimensions of abortion stigma were revealed in the current study.

## Methods

### The study sample

This study used in-depth interviews to provide insights regarding experiences of skilled providers associated with the practice of legal abortion provision. The study was conducted in Ghana’s capital city, Accra. A purposive sample of 36 health providers working in maternal/newborn health was recruited from the national, regional and district hospitals and from urban health centres. Interviews were recorded after informed consent was given. Informed consent involved a study information sheet being given to all prospective participants to take away and consider, then a follow-up call a few days later to see whether they were willing to participate. Of the 41 participants asked to participate, 36 were willing and 5 refused because they were not comfortable discussing the topic or did not want to be associated with it. All the interviews were conducted in English and took place between November 2006-July 2007.

Health professionals were purposively sampled from a range of public and private facilities, staff being selected with the help of the unit/facility heads. The three large hospitals in Accra (the teaching, regional and district hospitals) were selected since these see referred cases from across the country and capital city; these workers have substantial knowledge, exposure and experience of abortion. Midwives, rather than nurses, were included because it is they who staff the RCH units providing antenatal, post-natal and family planning services where women in need of reproductive health care services most commonly present. In addition, three private sector establishments were included to give a view of practices in the private sector. The selection of the private practitioners was based on foreknowledge by the first author and the fact that they were active in providing legal abortion services and/or advocating for provision of comprehensive abortion care. A group of private midwives were met after one of their general meetings, and on the recommendation of their leader, one member who was willing to be interviewed was included in the study. The pharmacists were included in the study because in Ghana studies have shown that community pharmacy shops are the first point of call when women have an unwanted pregnancy since abortion services are not openly available in public hospitals and private clinics are very expensive. Also, some pharmacy shops are known to sell abortifacients (e.g., Cytotec or Misoprostol) to women seeking abortion although this is not an over the counter drug. Women do not have much difficulty obtaining medications such as Misoprostol for medical abortion; if one pharmacy shop declines to dispense the medication to them without prescription, they will try other shops until they get it.

### Methods and analysis

A qualitative research approach (in-depth interviews) was chosen to capture rich textual descriptions with exemplars to illuminate the subjective meanings of phenomena (legal abortion provision) being studied. This provides a context in which to place findings to portray the real world of those studied [25].The words used by participants or observations made were analyzed to describe and interpret their meaning in order to answer the research question [26]. Questions to participants focused on areas including knowledge and perceptions of providers on the abortion law and policy, attitudes towards abortion service provision and, barriers to provision of services. Specifically, the questions were both open ended and semi structured and explored feelings, attitudes and beliefs of participants [27]. The topic guide was pre-tested among five doctors and three midwives, then finalised. Topics included: understanding of and attitudes to the law and policies relating to provision of legal abortion in Ghana; views and attitudes towards abortion practice in general; direct or indirect experiences of legal abortion provision; experiences or views on stigma associated with legal abortion provision.

Data were managed using NUD*IST software and Framework Analysis was used for analysis after code clusters from the software had been exported from the software into excel sheets for manual analysis [28]. One researcher (PA) analysed all interviews in depth in consultation with two other researchers (in particular SM). Data collection and data analysis took place concurrently and field notes were recorded to capture non-verbal communication and augment data interpretation. All but two interviews were recorded using a digital recorder and transcribed verbatim. For the two participants who did not allow their interviews to be recorded, notes were taken during the interview and field notes were written after the interview. Transcribed data were read several times to identify recurring themes. Themes were recorded and scrutinized for patterns. Based on identified patterns, the themes were grouped in a hierarchical manner. A code frame was developed and used to index the entire data set. Following indexing, all data under a sub-theme were pulled together and descriptive accounts were written on each sub-theme.

Trustworthiness was supported through triangulation of data sources and methods. Data sources (Obstetricians, midwives and pharmacists) and data collection methods (in-depth interviews and document analysis) were used to confirm and ensure completeness of the findings. The researcher’s prolonged field engagement (nine months) and checking the correctness of findings with participants supported credibility. Transcripts and the manuscript were reviewed by peers with whom the research was designed and discussed throughout the process. Before field work, the researcher recorded her thoughts about the research topic to minimize biases that could interfere with data interpretation. This act of reflexivity made the researcher aware of how her values and attitudes may influence interpretation of the research findings. The self-reflective position enhanced the credibility of the findings [29–31].

Ethical approval was granted by the Ethical Review Committees of the London School of Hygiene and Tropical Medicine (UK) and the Ghana Health Service. All participants provided written informed consent. Every effort was made to protect their identities.

## Results

Demographic characteristics of participants are presented in Table 1. Participants included nurse-midwives, obstetrician/gynaecologists and pharmacists ranging in age from 28 to 70 years with the pharmacists being the youngest on average. There were 17 men (mostly obstetrician-gynaecologists) and 19 women (mostly nurses). All had at least five years of experience at work with some having several decades; all qualified at least ten years before. Some pharmacists and doctors had received training overseas while only one nurse-midwife had.

**Table 1:** Demographic characteristics of participants (*n* = 36)

Demographic characteristics & participants	Age range	Sex		Years at Work	Av years of education	Training outside Ghana	Religion	
		Male	Female				Christian	Muslim
Obstetrician/gynaecologists *n* = 15	38–70	14	1	8–25	20	5	13	2
Nurse-Midwives *n* = 14	40–60+		14	10–30+	10	1	13	1
Pharmacists *N* = 7	28–49	3	4	5–20	14	4	7	0

A number of themes emerged from the interviews: first, a range of ways in which stigma creates barriers to provision of safe abortion care were revealed; second, detail on provider attitudes and experiences emerged. These are explored in turn.

### Barriers to provision of safe abortion care

#### Legal ambiguity creates perceived risk

Although most obstetricians regarded the law as liberal, a few described Ghana’s abortion law as restrictive in that gaps and inconsistencies existed within the law itself that created ambiguity and multiple interpretations; thus some were unwilling to perform a procedure that is not only stigmatised but may (by some people’s interpretation) be illegal. While for some, the inclusion of abortion in the Criminal Code renders abortion an offence, others regarded it solely as a health issue. Some obstetricians noted that the ambiguities in the law made it difficult to interpret and apply the law. One said:

*There is confusion within the profession. Because the law does state that only doctors may provide the service, many doctors are not sure; they are not certain whether they are covered or not. I have heard the opinion expressed that since I am a doctor, I can do it but supposing the police drag me to court; the MOH (Ministry of Health) is not going to support me to say it is part of the policy of the MOH that abortion under the conditions specified in the law may be provided by a qualified medical doctor; so I am left out there and because I am not certain what will happen to me, I would rather not do it.****Obstetrician/gynaecologist***

#### Stigma creates reluctance to advertise or request abortion services

A nurse-midwife reported that in the health facilities even though areas such as the “dispensary” and “labour ward” are clearly labelled, there are no designated places for abortion and women do not know where to go when they arrive and are afraid or shy to ask so they resort to clandestine abortions from unskilled providers:

*I think people do not know they can go to the hospital for safe abortion. They think they will not have it done.... This is why they go to have unsafe abortions. If they walk into a hospital and they are lucky and they find a nurse who will direct them; because it is not clearly written where they should go, they keep asking … she is shy, she does not want anybody to know her intentions so she probably asked one person and maybe the person does not want to hear about abortion and then she gets insults that will make her problem worse so she goes to a quack for an abortion.****Nurse/midwife***

One obstetrician noted that due to the intensity of stigma that marks those associated with abortion in Ghana, the word *‘abortion’* should be removed completely from any location where abortions are procured. He suggested the name *‘Comprehensive Reproductive Health care Centre’* and that *‘comprehensive abortion care’* in the policy document be reframed, and the term *‘abortion’* omitted.

#### Low quality of care and lack of recognition of abortion services

Reluctance to advertise abortion services helps perpetuate poor quality of care and lack of recognition of available services. Hospital administrators and heads of departments who are not in favour of providing abortion services will neither facilitate staff training nor procure manual vacuum aspiration (MVA) kits to enable skilled providers to offer abortion services. Some administrators will not even discuss the topic or attend meetings where abortion will be discussed. The reason given is that they hold positions in religious institutions and do not want to be seen to be associated with the topic. Others told doctors who requested privileges to conduct abortions that they do so at their own risk. One described his encounter with an administrator:

*There were challenges with abortion; it’s a moral issue so if you have leaders who morally are against abortion, then it becomes difficult. I recollect being told that, ‘I will not allow that nonsense to be performed in my health facility’ *. ***Obstetrician/gynaecologist***

Due to barriers to providing care in public hospitals, some will perform abortions in private clinics. The fees charged for abortions in private hospitals and clinics are expensive to some women. One doctor said:

*In this part of the world where people oppose abortion, safe abortion services are very expensive. It’s a major income generating thing…. [for one who says] “for something that I am doing that people don’t like and since I am doing it, you have to pay so much”… that service provider is just as bad as the person who opposes abortion. Because he says: ‘I will punish you by letting you pay with your life, and another says I will punish you by letting you pay so much’… we are in a society which feels that a person with an unwanted pregnancy deserves some punishment.****Obstetrician/gynaecologist***

#### Provider attitudes stigmatise clients coming for abortion

Attitudes of some providers, especially nurse-midwives frighten women who seek abortion. One observed:

*the health personnel – the way they look at abortion…it is a problem. It is part of the cause of unsafe abortion. They see you as a criminal if you go for abortion…the service providers’; they are very hostile, not friendly towards people due to their religious backgrounds –“abortion, who told you that in this place you can do abortion?” So, they scare the clients away.****Nurse/midwife***

Some providers opposed to abortion stated that women requiring abortions are promiscuous and sinful only seeking abortion to avoid the shame of having illegitimate children. One said:

*I feel we are giving license for women to have sex before marriage… they should not go into sex when they are not ready to have children… […] I won’t support those who say centres should be opened for legalized abortion.… Me, I am against abortion. Wait until you are married and when you are pregnant you wouldn’t want to abort it because the man married you to have children for him.****Nurse/midwife***

Community pharmacies are usually the first point of call for some women who need abortion services. Some pharmacies sell medications that can cause abortion without prescription. This was the concern of a pharmacist who said:

*I had problems with that. My personal sentiments are that all OTC (over the counter) drugs that could cause abortion be controlled; Menstrogen, Cytotec, Gynaecosid. Cytotec(Misoprostol) is all over the market. You insert some as pessary, swallow some and you are free. Lots of abortions are going on, unsafe and safe. There are pharmacists who help with counselling in their communities. Somebody wants to buy it; you know that she wants it for abortion. It should strictly be by prescription. In the communities prescriptions are not used****. Pharmacist***

Other pharmacists made the following observations which portrayed their varied sentiments and attitudes towards abortion service provision and the multifaceted religious, moral, legal, political, economic and sociocultural dimensions abortion provision evokes:

*Personally, I don’t indulge in such things…I won’t encourage the person to go and terminate. God says thou shall not kill; if God says don’t do this, it is for your own good! There are some pharmacies which don’t even stock some of these products…patients sometimes abuse them…some of these drugs used in terminating pregnancies like gynaecosid, menstrogen and cytotec…sometimes you may end up terminating a pregnancy by trying to help…If I am in such an environment, I will tell you to see a doctor…we refer them****.Pharmacist***

One pharmacist thought that they should be allowed to perform medical abortions in the community pharmacies because they are closer to the women in need of such services and are also the first point of call. He thought this would also give them extra income since he thought they were not well paid:

*If you use it and it doesn’t work, you refer the patient. It’s all economic; the obst and gynae doctors do it for economic reasons. They are making money…that is their source of income. There should be a sort of collaboration between the doctor and the pharmacist because they are both doing it [abortion]. We can’t think that it is not going on…something must be done for the benefit of the woman because most of these women see the pharmacists. They are the first people they see. There are few hospitals and clinics in the community but there are so many pharmacists****,****the point of contact of every one of them [women] is the pharmacist.****Pharmacist***

#### Stigmatising socio-cultural and religious norms shape provider attitudes and practices

##### Attitudes towards abortion providers and their clients

Most participants noted that Ghanaian culture considers abortion to be reprehensible. Children are highly valued and considered as gifts from God. Thus women who do not bear children in Ghana are frequently stigmatized. Participants reported that many people consider abortion as a taboo and a deeply shameful act, not worth discussing in public. Those who procure or provide abortions may be considered immoral or even murderers. A nurse-midwife said:

*I feel it’s butchering human beings into pieces…there’s a silent cry inside the womb… the voiceless are being murdered silently… I fear abortion… an interruption of life! It’s against the rule of the creator.… They [abortion providers] are destroying human life… the Bible says in Jeremiah 1:4-5-“The word of God came to me saying before I formed you in the womb, I knew you; before you were born I set you apart; I appointed you as a prophet to the nation”…the word of God is so clear about us human beings before we were formed in our mother’s womb so- to- destroy… we’re violating the rules of the creator.****Nurse/midwife***

Another said all those who offer abortions are answerable to God on the day of judgement. She talked of God demanding the *‘blood’* and *‘life’* of all the foetuses killed by providers. With respect to religion one obstetrician noted:

*Abortion, sex, Adam and Eve… we are going back to the Bible… people are very uncomfortable about it [abortion]. It doesn’t however diminish the danger to women’s health and lives… I accept that people have a moral problem about abortion. Nobody likes it! I’m not talking because I like abortion, No! My first [option] is to prevent it…” Ghanaians are very religious so the churches have a pervasive influence in which they think that abortion is bad.****Obstetrician/gynaecologist***

Most nurse-midwives interviewed were worried about abortion, frequently referencing religious prohibition. One who had all her training in Ghana said:

*I will object to it. I will never do it… I wouldn’t want to offend my God. Because he says don’t do it. So I don’t want to do it. When you do it, your hands become bloody. The Bible tells us that when you do it you are killing.****Nurse/midwife***

##### How beliefs and attitudes affect provision of legal abortion

The strength of religious beliefs and moral views described above meant that most nurse-midwives would not undergo training for comprehensive abortion care though they would willingly train for post abortion care since a woman with abortion complications has already initiated the abortion herself. They are only caring for her so that she does not die from the complications. They see this as good practice but would usually counsel women against abortion. Others refer them without guiding them about where to go. Some also refer women to clinics where they know abortions are provided but experience some dissonance in doing so:

*In the Christian way, we are not to do abortion, this is a very difficult situation but my work is to prevent death, especially maternal death. So, when someone approaches me with such a situation, I will refer the client to see the doctor …after referring her, I reflect; if it is done for her, it means I have taken part****. Nurse/midwife***

Another nurse-midwife with additional education abroad believed that women and young girls should have access to safe abortion and advice on post abortion contraception. She said:

*These girls are getting pregnant at a tender age and they know they cannot afford to look after the children so they go to the backstreet abortionist and they abort for them and they end up dead, pelvic infections… I think what can be done is to make safe abortion available… that is very controversial… If safe abortion is made available and people are told where they can get it, it may help…because they’re dying; maternal deaths from abortion is over 30-35 % and these are young girls…I don’t have anything against it so long as that person needs it the person should be referred to the right place....Instead of people dying it will be better to save them; once you save them you educate them properly.****Nurse/midwife***

Only a few nurse-midwives appeared to be highly knowledgeable about international declarations and recommendations regarding human rights issues:

*Everybody has a right … so if somebody comes and the person qualifies to have an abortion, it is a right. We should not deny them their right…most of them do not even know their rights; that is why they go to the quacks.****Nurse/midwife***

Several of the obstetricians had studied abroad at some point, or had exposure to international debate on safe-abortion. Most physicians said they would provide counselling and abortion services if necessary, but some acknowledged only as a last resort:

*Well, as obstetrician/gynaecologist, I don’t induce abortion but if you come, I will refer you if you have a genuine problem.****Obstetrician/gynaecologist***

One said he would do it only when there is no other skilled provider and a woman is in danger of imminent death. A few doctors declared themselves conscientious objectors and would not provide services themselves but were willing to refer to colleagues (or clinics). One obstetrician (a Catholic) would neither refer women nor provide the service himself. As a group, though, there was support for providing comprehensive abortion care among the obstetricians, many cited reasons such as their mandate from international conventions and in loyalty to professional oaths.

Despite many obstetricians’ willingness to provide abortion services or refer women for such services, they were concerned about stigma by association, noting that providers as well as women seeking abortion are labelled:

*“It is because of the notion people have about abortion. If you do abortion, you are evil… we have all been brought up that way; thinking negatively about abortion…”*

*“If you are seen to be doing abortions, you are labelled. It will take someone who is strong willed and immune to what people say to offer abortions in a public health facility. Doctors cannot openly speak for provision of safe abortion for fear of being labelled abortionists.”****Obstetrician/gynaecologist***

Another noted denial by obstetricians that they did in fact perform abortions:

*I have talked to many people I know who do abortions… put them a direct question, whether because he believes that women have a right to abortion he will terminate pregnancy, he will say no. Why is it that people don’t want to be known that they are doing abortions?…They don’t want to be known as abortionists.****Obstetrician/gynaecologist***

Doctors who do not want others to know that they provide abortions in public hospitals either do so but do not record them or record but misclassify them:

*Sometimes doctors hide behind the fact that for medical reasons they can perform abortions and do it en mass…. They will say ‘diagnostic D and C’ but, you and I know that there is nothing diagnostic about it… in actual fact it’s just an abortion, TOP (termination of pregnancy****). Obstetrician/gynaecologist***

#### Researcher reflections

One nurse-midwife initially agreed to be interviewed but each time she was contacted she had an excuse for postponing the interview. Although she was in a leadership position with much experience treating abortion complications she explained that she did not want to think or talk about abortion.

A member of an abortion research team and who had attended several conferences and meetings about the topic said she did not have any information to share on abortion when contacted for an interview. Although, she granted the interview, she was very succinct and guarded.

As the instrument for a qualitative investigation and due to the first author’s prolonged engagement in the field, she became an object of stigmatization through gossip. This did affect the researcher’s interpretation of data and future willingness to generate important evidence for safe care. In the hospitals where data were collected the researcher noted colleagues expressing disapproval with whispers and facial expressions. Others declined interviews. One student noted: *“Mrs. A, I thought you were a Christian, why are you undertaking such a study?*” Another colleague said, *“I don’t envy you one bit for the kind of study you are undertaking; good luck”*.

A number of people who were working in positions where the researcher knew they would be able to provide valuable information simply declined participation. One said she did not like the topic. There were times when the researcher felt isolated. Two potential participants who agreed to be interviewed appeared hostile. One would not allow the interview to be recorded and the other appeared furious. The researcher herself felt stigmatized.

## Discussion

Our study has documented a variety of ways in which the provision of abortion and those providing legal abortion services are stigmatised by providers themselves and by the wider community. Abortion stigma is “a negative attribute ascribed to women who seek to terminate a pregnancy that marks them, internally or externally, as inferior to ideals of womanhood” [32]. Women who are known to have participated in abortion in Ghana are generally not respected. They may be tagged as murderers and sometimes ostracized from their communities leading to “loss of status”, “labelling” and “separation” [16]. This has been the usual focus of abortion-stigma research and as we have seen in our study many providers share these stigmatising attitudes. Stigmatization also affects health providers who provide abortions as well as people who support women seeking abortions including advocates and researchers [33, 34]. Goffman [15] refers to stigmatization of others besides women seeking abortion as “courtesy stigma”; others refer to it as “affiliate stigma” [35]. Our study has clearly highlighted this affiliate stigma, which was recognised, experienced and sometimes perpetrated by the providers in our study. Studies have shown how such stigma is manifest in the treatment of both women and their providers, omission of the topic in policy documents or curricula and limited clinical training opportunities that might expose providers to different ways of thinking [36]. Our study additionally shows how this stigma affects providers’ own practice in terms of being reluctant to make it clear that safe, legal services are available at their facility, being reluctant to refer, conducting only post-abortion care and refusing to go on training. Some abortion providers charge high fees due to the stigma they are subjected to for carrying out the procedure; women who cannot afford the high fees resort to low cost methods which may be unsafe, including over the counter use of non-licensed drugs. Thus overall, stigmatization exacts a heavy toll on access to comprehensive abortion care, safety of the procedure, training, policy, practice and research in the area. It also promotes unsafe abortion.

The lack of resources including providers who are trained and willing to provide services have also been highlighted [14, 37, 38]. Some obstetricians in this study believed that lack of resources for abortion services was due to political ambivalence and lack of commitment on the part of those in authority. Restrictive laws are characterized by stigmatization, hence some have argued that legalization of abortion will reduce the stigma [12]. Most African cultures perceive abortion as “dirty work” thus legalization will not make it clean so many prefer not be associated with the procedure [34]. Our findings further suggest that the perceived ambiguity of the law in Ghana exacerbates the reluctance of otherwise willing providers, who are unsure whether they would receive institutional support for (legally) providing safe-abortion services. It must also be noted that legalization of abortion in the United States did not wipe away the stigma and service-providers frequently come under attack [33]. Clearly providers are keen to avoid any stigma by association with abortion services and our findings are consistent with those of other investigators who documented that abortion activities were conducted clandestinely and with a surrounding culture of silence [14].

In this study, nurse-midwives appeared particularly judgemental and their views reflected the cultural/religious values of their surrounding communities; they either said they did not want to or would refuse to provide abortion care. Ghanaian midwives are typically socialized in a cultural milieu that denounces abortion so they have inculcated in their minds that abortion is sinful. In contrast, doctors were less judgemental and more influenced by their professional judgements than religious arguments. This seemed to be related to their greater exposure to other settings (often training in western countries for many years). The impact of training and educational levels on promoting favourable provider attitudes towards reproductive and sexual health supports findings previously reported [39]. Nevertheless, obstetricians believed they were labelled when they conducted abortions; this finding supports the work of other investigators [14].

Some obstetricians were less concerned about community attitudes and continued to conduct abortions because they believed it was their professional obligation. In a qualitative study, those who conducted abortions considered themselves as “brave people” doing important work [36]. They put more emphasis on the positive aspects of abortion, while avoiding those deemed negative. Given the discrediting and devaluing description of stigma in Goffman’s seminal work [15] and the more recent theories that followed, it takes a determined and adequately motivated provider to accept stigma associated with abortion and to provide this service. Whilst some obstetricians and nurse-midwives in our study spoke of ethical dilemmas they faced regarding their professional obligations and religious beliefs, it has been reported elsewhere that professional difficulties with anti-abortion colleagues and burnout were some of the effects of stigma associated with abortion [33]. In Ghana, the religious milieu accentuates this phenomenon while in the United States; the formidable anti-abortion context puts those who provide abortions under pressure.

### Implications for policy and practice

Our findings have implications for education, practice, policy and future research. Values clarification workshops for all health providers, both in - service and pre - service could be implemented to enhance non-stigmatising attitudes towards women who have had abortions and who may have to contend with medical and psychological consequences of their choices. Values clarification is an intervention that transforms attitudes and behaviours of health providers to be less judgemental and more positive towards abortion services [40]. It is a useful strategy the Ministry of Health/Ghana Health Service and Nursing and Midwifery Council could explore for use in exposing nurse-midwives and other skilled providers to alternative, evidence-based views. The Ghana Health Service can create awareness in health providers or sensitize them to the negative effects of stigma, particularly for the women seeking care. This could help all health providers alter negative or unhelpful attitudes and behaviours towards women who seek abortion and those can safely provide and care for them.

The Ghana Health Service [6] standards and protocols for comprehensive abortion care, which does clarify the ambiguities in the law and explains the roles and responsibilities of health providers offering, should be widely disseminated to all practicing and student health providers including nurse/midwives and obstetrician/gynaecologists so they have an opportunity to understand their professional responsibilities and that there is institutional support for implementing them. Relevant and clear policies need to be developed by the Ministry of Health to ensure that women who procure reproductive health services and skilled providers who offer such services are not harassed. Consistent with the WHO definition for those who conduct safe abortions or care for women who have had one, we used the term “skilled provider” for health providers who have specific training in this area. Further research could focus on identifying pragmatic strategies to deal with stigma in a meaningful way.

## Conclusions

Stigma constitutes an overarching impediment for abortion service provision. It affects providers’ attitudes and practices in a variety of ways through perceived “stigma by association”. It affects health providers offering such services and even researchers who study the subject. Exposure to wider debate and education seem to influence attitudes therefore values clarification training may prove useful for promoting less stigmatising views among providers, especially in the nurse-midwife cadre. Proper dissemination of existing guidelines and overt institutional support for provision of safe services also needs to be rolled out to minimise the effects of stigma on providers.

## Abbreviations

D&C: Dilation and curettage
D&E: Dilation and evacuation
MVA: Manual vacuum aspiration
NGO: Non-Government Organisation
OTC: Over the counter
WHO: World Health Organisation
